# Comparison of Various Assays of Antioxidant Activity/Capacity: Limited Significance of Redox Potentials of Oxidants/Indicators

**DOI:** 10.3390/ijms26157069

**Published:** 2025-07-23

**Authors:** Paulina Furdak, Kacper Kut, Grzegorz Bartosz, Izabela Sadowska-Bartosz

**Affiliations:** 1 Laboratory of Analytical Biochemistry, Institute of Food Technology and Nutrition, Faculty of Technology and Life Sciences, University of Rzeszow, 4 Zelwerowicza Street, 35-601 Rzeszów, Poland; paulinaf@dokt.ur.edu.pl (P.F.); kkut@ur.edu.pl (K.K.); gbartosz@ur.edu.pl (G.B.); 2 Doctoral School, University of Rzeszow, 16C Rejtana Street, 35-959 Rzeszów, Poland

**Keywords:** ABTS, antioxidants, Cupric Ion Reducing Antioxidant Capacity, 2,6-dichlorophenolindophenol, ferricyanide, Ferric Reducing Antioxidant Power, *o*-phenanthroline, Oxygen Radical Absorbance Capacity, TAC, Trolox

## Abstract

Assays of total antioxidant capacity (TAC) of complex materials bring no information on the composition of antioxidants present in a sample. As the thermodynamic condition for a redox reaction is that redox potential of the oxidant must be higher than that of a reductant (antioxidants), it seemed to be of interest whether it is possible to estimate the content of antioxidants of various ranges of redox potentials using a set of assays employing oxidants/indicators of different values of redox potentials. Antioxidant activities of nine antioxidants and TAC of an aqueous garlic extract were estimated using nine assays of E_o_′ of oxidants/indicators ranging from 0.11 to 1.15 V. The antioxidant activities were expressed in mol Trolox equivalents/mol compound. The thermodynamic conditions made some antioxidants unreactive with indicators of sufficiently low E_o_′, but otherwise, no dependence between the antioxidant activities and redox potentials of oxidants/indicators and reactivities of antioxidants was observed. TAC of the garlic extract did not show any regular dependence on the redox potential of the oxidant/indicator, being the highest in the test of 2,2′-azinobis-(3-ethylbenzothiazoline-6-sulfonate) radical (ABTS^•^) decolorization. These results indicate that kinetic factors play a primary role in determining the antioxidant activities of antioxidants and TAC in various assays.

## 1. Introduction

The antioxidant activity/capacity is a measure of the ability of the studied compound/material to react with oxidants. One of the basic problems in studies of the antioxidant activity of various compounds and total antioxidant capacity (TAC) of complex materials, such as food products or physiological fluids, is the diversity of results obtained by different assay methods, making comparisons between results of various authors difficult or even impossible. Such differences may arise even within the same method, for example, when different reaction times, various buffers, or temperatures are used. However, more important differences concern assays performed by different methods [1,2,3]. As an example, some values reported for the antioxidant activity of gallic acid can be provided. A value of 2.62 mol Trolox equivalents (TE)/mol was reported in the Cupric Ion Reducing Antioxidant Capacity (CUPRAC) assay [4]; 2.23 mol TE/mol [5] and 2.78 mol TE/mol [6] in the ferricyanide reduction assay; 3.21 mol TE/mol [7], 3.48 mol TE/mol [6], 4.33 mol TE/mol [8], and 4.73 mol TE/mol [9] in the ABTS^•^ reduction assay; 1.98 mol TE/mol [7], 2.94 mol TE/mol [10], 1.85 and 3.05 [6] in the Ferric Reducing Antioxidant Power (FRAP) assay; 3.86 mol TE/mol in the Fe(III)phenanthroline reduction assay [6] and 1.05 mol TE/mol in the Oxygen Radical Absorbance Capacity (ORAC) assay [7]. Various authors postulated thus to assay the antioxidant activities, especially of complex materials, by more than one method [11,12].

One of the main reasons for differences between results from various assays of antioxidant activity/TAC seems to be the difference in the redox potential of oxidants used in these assays. The redox potential of compounds reducing an oxidant must be lower than that of the oxidant; the lower the redox potential of the oxidant, the more limited is the pool of potentially reactive antioxidants. It might appear that a set of antioxidant assays based on oxidants/indicators of different redox potentials could provide information on the contribution of antioxidants with various redox potentials to TAC, thereby making the analysis of TAC more insightful.

This study was aimed at a verification of this hypothesis using several assays of antioxidant activity/TAC: Fe(III)/*o*-phenanthroline reduction, ORAC, FRAP, CUPRAC, 2,2′-azinobis-(3-ethylbenzothiazoline-6-sulfonate) radical (ABTS^•^) decolorization, ferricyanide reduction, 2,6-dichlorophenolindophenol (DCIP) reduction, and Methylene Blue reduction assays to estimate antioxidant activities of nine antioxidants and TAC of aqueous extract of garlic *Allium sativum* L. The list of antioxidants included Trolox, commonly used as a standard in antioxidant activity assays, ascorbic acid, glutathione, NADH, gallic acid as an example of a polyphenol, allicin—the most reactive organosulfur compound of garlic—and three nitroxides, 2,2,6,6,-tetramethylpiperidine-1-oxyl (TEMPO), 4-hydroxy-TEMPO (TEMPOL) and 4-amino-TEMPO (TEMPAMINE).

## 2. Results and Discussion

**Figure 1:**
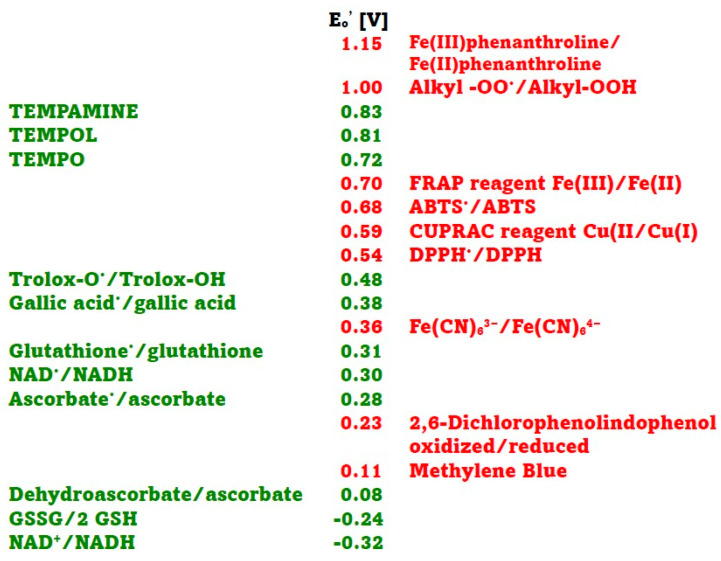
Standard redox potentials at pH 7 of selected oxidants (red) and antioxidants (green) used in antioxidant activity assays. Data taken from [5,14,18,19,20,21,22,23,24].

In this study, several assays of antioxidant activity/capacity were employed, representing a range of redox potentials of oxidants/indicators and antioxidants. The redox potentials of the oxidants/indicators and antioxidants used are shown in Figure 1. In the antioxidant activity essays employed, the Fe(III)/Fe(II) phenanthroline redox system had the highest standard redox potential. Values of 0.82 V [13] and 1.06 V [5] have been reported for this redox couple, but the value of 1.15 V [14] is most commonly cited. The alkyl peroxyl radical/alkyl hydroperoxide couples, which are the oxidants in the ORAC assay, have redox potentials in the range of 1 V (0.77–1.44 V, depending on their structure) [14,15,16].

The standard redox potentials E_o_′ of the Fe(III)-2,4,6-tri(2-pyridyl)-s-triazine (TPTZ)/Fe(II)TPTZ (ca 0.70 V) and ABTS^•^/ABTS (0.68 V) couples are similar [17]. The standard redox potential of the Cu(II)/neocuproine/Cu(I)neocuproine couple is 0.59 V [5], and that of 2,2-diphenyl-1-picrylhydrazyl radical (DPPH^•^)/DPPH couple is 0.537 V [18]. The standard redox potential of ferricyanide/ferrocyanide couple is 0.36 V [5], and those of oxidized 2,6-dichlorophenolindophenol (DCIP)/DCIP and oxidized Methylene Blue/Methylene Blue couples are 0.228 and 0.011 V, respectively [19].

The standard redox potentials of the dehydroascorbate/ascorbate, oxidized glutathione/reduced glutathione, and NAD^+^/NADH couple are 0.08 V, −0.24 V and −0.32 V, respectively [20]. However, these values concern two-electron reactions while most of the reactions in standard antioxidant assays are one-electron assays, so redox potentials for one-electron reactions should be considered. The standard redox potential of the ascorbyl radical/ascorbate couple is 0.282 V, and that of the dehydroascorbate/ascorbyl radical couple is −0.174 V [14]. The standard one-electron redox potential of the glutathione cysteinyl radical/glutathione determined by potentiometric titration at pH 7.4 was found to be 0.31 V [21], while that of the oxidized glutathione/glutathione cysteinyl radical couple is −1.5 V [14]. The one-electron potential of the NAD^•^/NADH redox couple is 0.30 V [22]. The standard redox potential of Trolox radical/Trolox couple is 0.48 V [9,14] and that of gallic acid radical/gallic acid is 0.377 V [23]. Standard redox potentials of respective oxoammonium ions/TEMPO, TEMPOL and TEMPAMINE couples were reported to be 0.722, 0.810 and 0.826 V, respectively [24]. We were unable to find data on the redox potential of allicin.

The antioxidant activities of nine antioxidants obtained by various assays, expressed in Trolox equivalents (TE), are shown in Table 1. In the assay of DCIP reduction, the absorbance decrease was a net result of DCIP reduction and re-oxidation of the leuco-dye. The reduction of DCIP by ascorbate and glutathione was maximal after 10 min; longer incubation decreased the extent of reduction, apparently due to re-oxidation. In contrast, the reduction of DCIP by NADH and Trolox was maximal after 60 min, and the measurement probably underestimated the extent of reduction due to the simultaneous reoxidation of reduced DCIP. In the Methylene Blue reduction assay, only ascorbic acid showed reducing activity. The garlic extract reduced 13.0 ± 3.4% of the dye while 1 mM ascorbate reduced 6.7 ± 2.1% of DCIP.

Allicin was found to show significant reactivity only in the ORAC assay in our hands. Only NADH, glutathione, ascorbic acid, and gallic acid reacted in the DCIP reduction assay. This was expected for the first three compounds as their two-electron redox potentials are lower than that of the DCIP redox couple. The reactivity of gallic acid in the DCIP reduction assay is surprising, as the standard redox potential of the gallic acid radical/gallic acid system is significantly higher than that of the DCIP redox couple (Figure 1). Similarly, the reactivity of nitroxides in the ferricyanide reduction assay is hard to explain, taking into account the reported values of their redox potentials. The possible explanations could include: (i) erroneous values of the reported redox potentials, (ii) one-electron instead of two-electron reactions or vice versa, or (iii) differences between the standard and real redox potentials of the reacting systems. It should be remembered that the standard redox is defined for a system in which the activities or concentrations of the oxidized and reduced forms of the compound are the same and equal to 1 M. In other situations, the redox potential of a system is determined by the Nernst equation.

$$E_{h}=E_{o}\;+\;\frac{RT}{nF}\ln\frac{\left[ox\right]}{\left[red\right]}$$

where *E_h_* is the redox potential of a redox couple at any ratio of the oxidized and reduced form of a compound; *E_o_* is the standard redox potential of the redox couple; *R,* gas constant; *F*, Faraday constant; *T*, temperature; *n* is the number of electrons involved in the reaction; and [*ox*] and [*red*] are the activities or concentrations of the oxidized and reduced, respectively, forms of the compound.

The real difference in the redox potentials of an antioxidant (present initially almost entirely in the reduced form) and an oxidant/indicator (present initially almost entirely in the oxidized form) may differ significantly from the standard values. It results from the Nerst equation that when >99% of the antioxidant is in the reduced form, the actual redox potential of the reducing system is lower by about 0.1 V than its standard redox potential; conversely, when >99% of the oxidant is in the oxidized form, the actual redox potential of the oxidizing system is higher by about 0.1 V than its standard redox potential. Under such conditions, the reaction is thermodynamically possible but may lead to only partial reduction of the oxidant, until reaching thermodynamic equilibrium. Apparently, this might be the case for DCIP reduction by Trolox; in this assay, one Trolox molecule reduced only 0.28 molecules of DCIP on average, although in most assays, the reactivity of Trolox is conditioned by donation of two electrons by one Trolox [25].

Moreover, the concentration of oxidant/indicator can significantly affect the stoichiometry of the reaction, which decreases with increasing concentration of the oxidant/indicator [9,26].

In some cases, antioxidants showed low reactivity. This was the case for glutathione in the assays of Fe(III)phenanthroline reduction, FRAP, and ferricyanide reduction. Allicin was weakly reactive in the Fe(III)phenanthroline reduction assay, though quite reactive in the ORAC assay. It should be taken into account that a lower value of the redox potential of an antioxidant with respect to an oxidant is only a necessary thermodynamic condition but says nothing about the rate of the reaction, which may be slow enough to escape detection in a short assay.

The low reactivity of glutathione in the DPPH^•^ decolorization assay may be related to its lower solubility in methanol as compared with aqueous media; it was demonstrated that glutathione reactivity in this assay is much higher in a methanol buffer than in methanol medium [27].

Interestingly, all nitroxides studied (TEMPO, TEMPOL and TEMPAMINE) and allicin showed high activities in the ORAC assay, while other antioxidants, including NADH, glutathione and ascorbic acid, exhibited relatively low activities. It supports the view that the ORAC method is chemically more relevant to chain-breaking antioxidant activity [15]. Nitroxides are known to react with peroxyl radicals and act as chain-breaking antioxidants [28,29,30]. A similar chain-breaking antioxidant activity has been reported for allicin [31,32].

**Figure 2:**
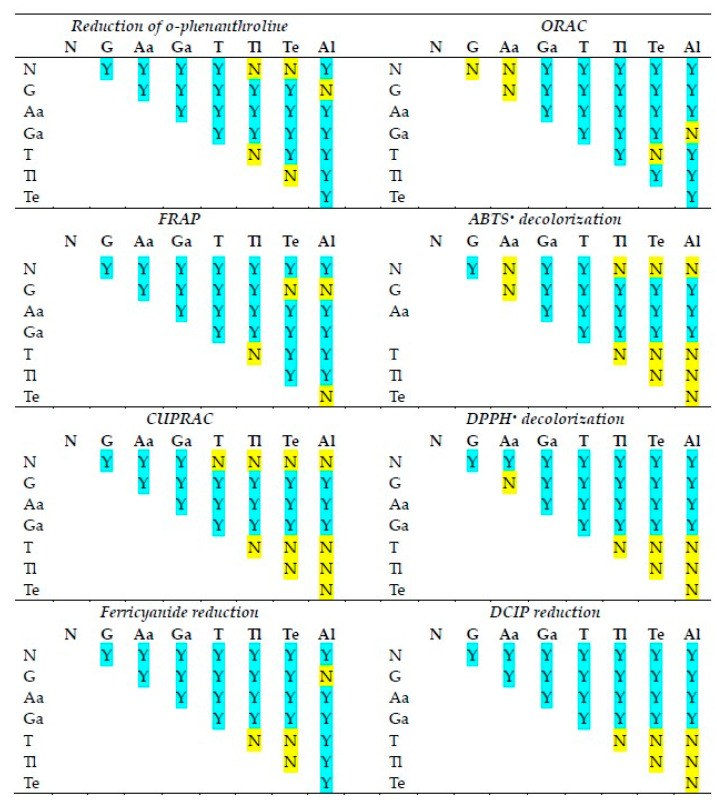
Statistical significance of differences between antioxidant activities of the antioxidants studied in various assays of antioxidant activity. N, NADH; G, glutathione; As, ascorbic acid; Ga, gallic acid; T, TEMPO; Tl, TEMPOL; Te, TEMPAMINE; Al, allicin. Y, statistically significant difference; N, difference not significant. One-way ANOVA with Tukey’s post-hoc test, *p* < 0.05.

**Figure 3:**
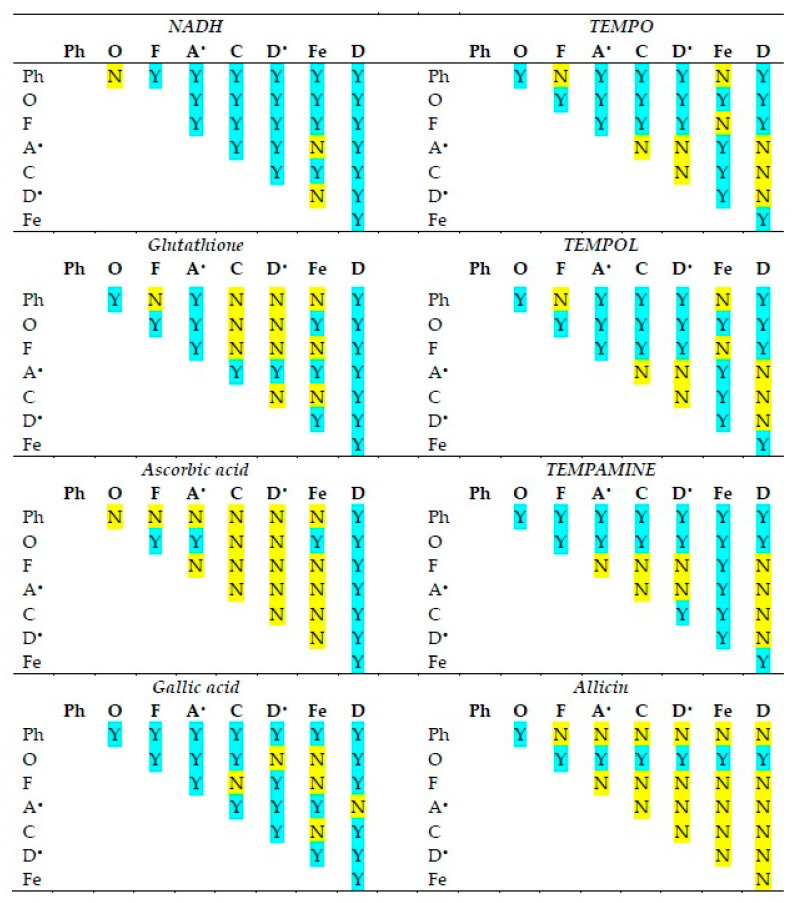
Statistical significance of differences between antioxidant activities of the antioxidants studied in various assays of antioxidant activity. Ph, *o*-phenanthroline reduction; O, ORAC; F, FRAP; A^•^, ABTS^•^ decolorization; C, CUPRAC; D^•^, DPPH^•^ decolorization; Fe, ferricyanide reduction; D, DCIP reduction. Y, statistically significant difference; N, difference not significant. One-way ANOVA with Tukey’s post-hoc test, *p* < 0.05.

The statistical significance of differences in the reactivities of the antioxidants studied in various antioxidant activity assays is shown in Figure 2. The statistical significance of differences in the reactivities of individual antioxidants in various antioxidant activity assays is compiled in Figure 3.

Although in all assays, there are cases in which the reactivities of antioxidants of neighboring standard redox potentials are not statistically different, in the majority of cases, the activities of various antioxidants differed from each other in all assays.

The activities of individual antioxidants generally differed in various assays, although a lack of differences was found in many cases for ascorbic acid and allicin (the latter showing no significant activity in most assays).

A relation between the reactivity of various catechins and glutathione and their redox potential in the ABTS^•^ and DPPH^•^ decolorization assays was reported—antioxidants with lower redox potential values tending to be more reactive [21]. We checked the correlation between the standard redox potentials of the antioxidants used and their reactivities in various assays (Figure 3) and found the values of −0.22 for the assay of Fe(III)-phenanthroline reduction, 0.94 (*p* < 0.001) for ORAC, −0.52 for FRAP, −0.56 for the assay of ABTS^•^ decolorization, −0.46 for CUPRAC, −0.87 (*p* < 0.01) for the assay of DPPH^•^ decolorization, −0.36 for the assay of ferricyanide reduction, and −0.95 (*p* < 0.001) for the assay of DCIP reduction. Thus, although the expected negative correlation between the standard redox potentials of antioxidants and their reactivities was observed in the majority of cases, a statistically significant correlation was found only in some cases, and in one case (ORAC) it was, surprisingly, positive.

A correlation between the reactivities of individual antioxidants in various assays and the standard redox potentials of the oxidants/indicators was also checked. The correlation coefficients were: −0.65 (*p* < 0.05) for ascorbate, −0.67 (*p* < 0,05) for NADH, −0.56 for glutathione, −0.26 for gallic acid, 0.59 for TEMPO, 0.56 for TEMPOL, and 0.46 for TEMPAMINE. Most of the correlation coefficients were negative, but only two were statistically significant. Thus, there was a tendency toward an inverse relationship between the redox potentials of the oxidants/indicators of various antioxidant assays and the reactivities of antioxidants, but it was generally weak, and not universal.

The high values of antioxidant activity of NADH, glutathione, ascorbic acid, and gallic acid in the DCIP reduction assay are artifactual as they were standardized with respect to Trolox, which itself showed a weak reactivity in this assay. The number of DCIP molecules reduced by one molecule of NADH, glutathione, ascorbic acid and gallic acid was 0.89, 1.50, 1.37 and 1.22, respectively. This example points to a limited applicability of Trolox in assays involving indicators of low redox potential. Some authors prefer to use ascorbic acid as an antioxidant standard; the results presented in Table 1 show that, in general, ascorbic acid could be a good standard as well, applicable also to assays involving lower redox potential indicators. If recalculating the results for ascorbic acid as a standard, antioxidant activities and TAC values would not be very different than those using Trolox as a standard, except for the ORAC assay, for which the values of antioxidant activity and TAC would be twofold higher than for Trolox as the standard. However, Trolox can be used as a standard in assays performed in non-aqueous media, estimating hydrophobic antioxidants. Moreover, its solutions are more stable in comparison with those of ascorbic acid, which makes the use of ascorbic acid as a standard more troublesome and, if uncontrolled, may contribute to the variability of results.

**Figure 4:**
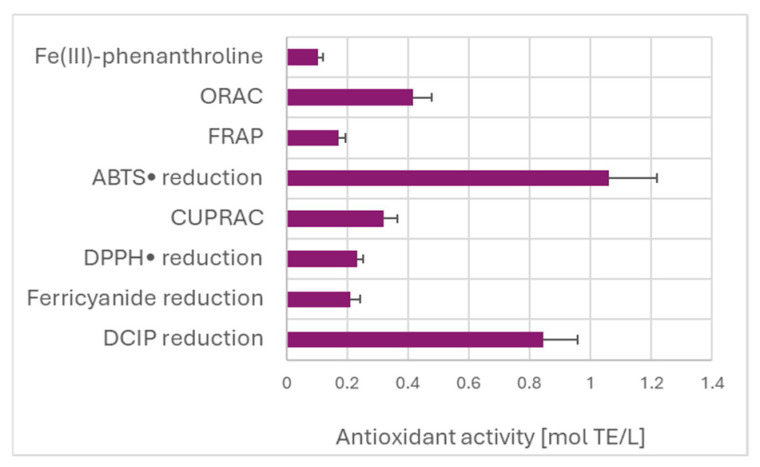
TAC of garlic extracts measured by different assays, referred to Trolox as the standard.

Estimation of TAC of the garlic extract by various assays did not show the expected dependence on the redox potential of the oxidants/indicators of the assays (Figure 4). The highest value of TAC was found for the ABTS^•^ decolorization assay. The high TAC value obtained in the DCIP reduction assay is due to the calculation with respect to Trolox, which is weakly reactive in this assay, as discussed before.

These results demonstrate that the idea of screening TAC for antioxidants from different ranges of redox potentials is not feasible and kinetic factors play a major role in the determination of the reactivity of various antioxidants in the assays of antioxidant activity/capacity.

## 3. Materials and Methods

### 3.1. Reagents and Materials

Ethanol (CAS no. 64-17-5, cat. no. 396480111, purity ≥ 99%), methanol (CAS no. 67-56-1, cat. no. 6219900110, purity ≥ 99.9%), glacial acetic acid (CAS no. 64-19-7, cat. no. JT9522-2), sodium acetate anhydrous (CAS no. 127-09-3, cat. no. BN60/6191, purity ≥ 99%), Methylene Blue (CAS no. 61-73-4, cat. no. 185480121, purity ≥ 82%), and fluorescein (CAS no. 2321-07-5, cat. no. F7505) were obtained from Avantor Performance Materials (Gliwice, Poland). 2,2′-Azino-bis (3-ethylbenzothiazoline-6-sulfonic acid) (ABTS; CAS no. 504-14-6, cat. no. 10102946001, purity ≥ 99%) was provided by Roche (Warsaw, Poland). Dimethyl sulfoxide (DMSO) (CAS no. 67-68-5, cat. no. D2438, anhydrous, ≥99.9%), (±)-6-hydroxy-2,5,7,8-tetramethylchromane-2-carboxylic acid (Trolox; CAS no. 53188-07-1, cat. no. 238813, purity ≥ 97%), phosphate-buffered saline (PBS) (cat. no. PBS404.200); TEMPO (CAS no. 2564-83-2, cat. no. 214000), TEMPOL (4-hydroxy TEMPO; CAS no. 2226-96-2, cat. no. 176141), TEMPAMINE (4-amino-TEMPO; CAS no. 14691-88-4, cat. no. 163945), L-ascorbic acid (CAS no. 50-81-7, cat. no. A0278), L-Glutathione reduced (CAS no.70-18-8, cat. no. G4251, purity ≥ 98%), 2,2-azobis(2-amidinopropane) dihydrochloride (AAPH; CAS no. 2997-92-4, cat. no. 440914), neocuproine hydrate (CAS no. 654054-57-6, cat. no. 121908), 2,4,6-tri-2-pyridinyl-1,3,5-triazine (TPTZ; CAS no. 3682-35-7, cat. no. T1253), copper(II) sulfate pentahydrate (CAS no. 7758-99-8, cat no. 209198), Trizma base (CAS no. 77-86-1, cat. no. T6066), 10-phenanthroline (CAS no. 66-71-7, cat. no. 131377, purity ≥ 99%), β-nicotinamide adenine dinucleotide (NADH) (CAS no. 606-68-8, cat. no. N8129, purity ≥ 97%), potassium hexacyanoferrate(III) (CAS no. 13746-66-2, cat. no. 208019, purity ≥ 99%), trichloroacetic acid (CAS no. 76-03-9, cat. no. T6399, purity ≥ 99%), and 2,6-dichloroindophenol sodium salt hydrate (CAS no. 1266615-56-8, cat. no. D1878) were provided by Merck (Poznań, Poland). Allicin (diallyl thiosulfinate, CAS no. 539-86-6, cat. no. HY-N0315, purity: 97.36%), 2,2-diphenyl-1-(2,4,6-trinitrophenyl)hydrazin-1-yl (DPPH; CAS no. 1898-66-4, cat. no. HY-112053, purity ≥ 99.13%), and iron(III) chloride (FeCl_3_; CAS no. 7705-08-0, cat. no. 451649, purity ≥ 99.99%) were provided by MedChemExpress (Monmouth Junction, NJ, USA). Phosphate-buffered saline (PBS; cat. no. PBS404.200), sodium dihydrogen phosphate (CAS no. 10049-21-5, cat. no. PM306.500, purity 98–103%), and sodium hydrogen phosphate (CAS no. 7782-85-6, cat. no. SPD579.1, purity 98–102%) produced by BioShop Canada Inc. (Burlington, ON, Canada) were purchased from Lab Empire (Rzeszow, Poland). Hydrochloric acid (CAS no. 7647-01-0, cat. no 115752837, 35–38%) was provided by Chempur (Piekary Śląskie, Polska).

Distilled water was purified using a Milli-Q system (Millipore, Bedford, MA, USA). Transparent flat-bottom 96-well plates (cat. no. 655101) as well as black-flat-bottom 96-well plates (cat. no. 655209) (Greiner, Kremsmünster, Austria) were used for the assays. Absorptiometric and fluorimetric measurements were conducted in a Spark multimode microplate reader (Tecan Group Ltd., Männedorf, Switzerland).

Fresh garlic (*Allium sativum* L.) bulbs grown in Spain were purchased in a local grocery shop. A portion of cut garlic cloves (about 5 g) was homogenized with phosphate-buffered saline (PBS; 0.9% NaCl in 10 mM sodium phosphate buffer, pH 7.4) in a proportion of 9 mL PBS/g garlic. The homogenate was centrifuged at 4000× *g* for 15 min and assayed immediately or frozen at −80 °C in small aliquots and used after thawing within no more than a month.

### 3.2. Fe(III)-Phenanthroline Reduction Assay

The protocol described by Özyürek et al. [33] was followed. The phenanthroline reagent was prepared by dissolving 0.3 mmol of FeCl_3_ and 1 mmol of 1,10-phenanthroline in 2 mL of 1 M HCl and diluting with de-ionized water to 100 mL. Aliquots of 200 µL of this reagent were pipetted into wells of a 96-well plate and added with various amounts of antioxidant solutions (1 mM or 5 mM) or the garlic extract. After 10 min of incubation at ambient temperature (21 ± 1 °C), the absorbance of the samples at 520 nm was measured.

### 3.3. The Oxygen Radical Absorbing Capacity (ORAC) Assay

A slight modification of the method of Ou et al. [34] was used. Briefly, increasing volumes (1–10 μL) of the antioxidants or the garlic extract were added to wells of a 96-well microplate containing 0.2 μM fluorescein and 50 mM AAPH (final concentrations) in a total volume of 200 μL of 50 mM sodium phosphate buffer, pH 7.4 (considering the extract volume). The decay of fluorescence was measured at 478 nm/520 nm every 90 s for at least 3 h at a temperature of 37 °C, and the sum of fluorescence intensities for all measurements was calculated for each sample. Per cent protection of the fluorescein fluorescence was calculated according to the formula

% protection = 100% [(S_sample_ − S_AAPH_)/(S_blank_ − S_AAPH_)]

where S_sample_ is the sum of fluorescence intensities for a studied sample, S_AAPH_ is the sum of fluorescence intensities of a sample containing AAPH and no antioxidants, and S_blank_ is the sum of fluorescence intensities of a sample containing no AAPH and no antioxidants.

### 3.4. The Ferric Reducing Antioxidant Power (FRAP) Assay

The method proposed by Benzie and Strain [35] was slightly modified. Briefly, increasing volumes of solutions of the studied compounds or the garlic extract were added to wells of a 96-well microplate pre-filled with 200 μL of the working solution composed of 0.3 M acetate buffer, pH 3.6 (10 volumes), 10 mM TPTZ in 40 mM HCl (1 volume), and 20 mM FeCl_3_ (1 volume), prepared immediately before use. After 30-min incubation at ambient temperature, the absorbance of the Fe^2+^–TPTZ complex was read at 593 nm.

### 3.5. ABTS^•^ Decolorization Assay

A modification [36] of the ABTS^•^ decolorization assay [37] was employed. Briefly, various amounts of antioxidants or garlic extract were introduced to wells of a 96-well microplate, each pre-filled with 200 µL of ABTS^•^ solution of absorbance 1.0 (at 734 nm) in a well of a 96-well microplate. The stock ABTS^•^ solution was prepared by overnight oxidation of 7 mM ABTS with 2.45 mM potassium persulfate (final concentrations). This stock solution was diluted with PBS so that 200 μL of the sample had an initial absorbance of 1.0 at 734 nm in a well of a 96-well microplate. This solution was added with various amounts of antioxidants or garlic extract. The drop in absorbance after 30-min incubation at ambient temperature, corrected for the absorbance decrease in a blank sample, containing ABTS^•^ solution without any additive, was read as a measure of the antioxidant activity.

### 3.6. The Cupric Ion Reducing Antioxidant Capacity (CUPRAC) Assay

A modified procedure of Özyürek et al. [4] was used. Briefly, 75 µL of 50 mM Tris-HCl buffer with a pH of 7.0 was mixed with 75 µL of 10 mM CuSO_4_, 75 µL of 7.5 mM 2,9-dimethyl-1,10-phenanthroline solution in ethanol, and various volumes of antioxidant solutions, garlic extract, or PBS to make up a volume of 75 µL. The total volume of the mixture was 300 µL. After 60-min incubation at ambient temperature, the samples were centrifuged, 200-μL aliquots of the supernatants were pipetted into wells of a 96-well plate, and their absorbance was measured at 450 nm against a reagent blank.

### 3.7. DPPH^•^ Decolorization Assay

The assay was performed by a modification of the method previously described [38]. The modification enabled elimination of the effect of turbidity appearing after the addition of the extracts to the methanol solution of DPPH^•^. Briefly, increasing volumes of antioxidant solutions or the garlic extracts and PBS to make a total volume of 20 μL were added to Eppendorf tubes containing 300 µL of 0.3 mM DPPH^•^ solution in methanol. The reaction was allowed to proceed for 30 min at ambient temperature, in the dark. The samples were centrifuged, 200-μL aliquots of the supernatants were pipetted into wells of a 96-well plate, and their absorbance at 517 nm was read. The absorbance decrease, with respect to samples containing DPPH^•^ solution added with PBS only, was a measure of the antioxidant activity.

### 3.8. Ferricyanide Reduction Assay

A slightly modified protocol of Vijayalakshmi & Ruckmani [39] was used. Various amounts of antioxidants or garlic extract were added to Eppendorf tubes containing 200 μL of 1% K_3_Fe(CN)_6_ and 100 mM phosphate buffer to make the final volume of 400 μL. The tubes were incubated in a shaker at 50° for 20 min. Then, 500 µL of 10% TCA was added, and the tubes were centrifuged (7000× *g* for 3 min). The supernatant (100 µL) was pipetted into wells of a 96-well plate, added with 100 µL of de-ionized water and 20 µL of 0.1% FeCl_3_ solution in 1 mM HCl. Absorbance of the product was measured at 700 nm.

### 3.9. 2,6-Dichloroindophenol Reduction Assay

Various amounts of antioxidants or garlic extract were added to wells of a 96-well plate containing 160 µM (final) DCIP and 50 mM phosphate buffer, pH 7.4. After 10- to 60-min incubation at ambient temperature, a decrease in absorbance was read at 596 nm.

### 3.10. Methylene Blue Reduction Assay

Various antioxidant solutions (1 mM) and the garlic extract (20 µL) were added to Methylene Blue solution in 50 mM phosphate buffer, pH 7.4, to make up the final volume of 200 µL in wells of a 96-well plate. The final Methylene Blue concentration was 40 µM. After 10–120 min of incubation at ambient temperature, the decrease in absorbance was measured at 665 nm. The results are expressed as a percentage reduction of Methylene Blue present in the samples.

### 3.11. Comparison with Trolox as a Standard

In all cases, Trolox was used as a standard. The results were calculated with respect to the antioxidant activity of Trolox and expressed in mol TE per mol of compound, except for the Methylene Blue reduction assay, where Trolox was not active. The TAC of garlic extract was expressed in moles TE/L of extract.

### 3.12. Statistics

The results are means ± SD of at least 3 experiments. The statistical significance of differences between antioxidants within individual assay methods of antioxidant activities and between various antioxidant assays for individual antioxidants was evaluated using one-way ANOVA with the post-hoc Tukey’s test, assuming *p* = 0.05. The calculations were performed using the XLSTAT program (Lumivero, Denver, CO, USA).

## 4. Conclusions

The present results demonstrate that, apart from the obvious thermodynamic conditions, kinetic factors govern the reactivities of antioxidants with various oxidants/indicators. This makes the idea of identifying the contributions of antioxidants of various redox potentials to the Total Antioxidant Capacity of biological materials or food products using a set of different antioxidant assays unfeasible.

## Figures and Tables

**Table 1:** Antioxidant activities [mol TE/mol] of the antioxidants studied in various assays.

Assay	NADH	GSH	Ascorbic Acid	Gallic Acid	TEMPO	TEMPOL	TEMP-AMINE	Allicin
Fe(III)-phenanthroline reduction	0.30 ± 0.04	0.006 ± 0.011	0.81 ± 0.06	3.11 ± 0.22	0.56 ± 0.09	0.43 ± 0.05	0.33 ± 0.04	0.0003 ± 0.0007
ORAC	0.32 ± 0.02	0.42 ± 0.05	0.50 ± 0.04	1.05 ± 0.09	1.59 ± 0.14	1.94 ± 0.22	1.48 ± 0.17	1.06 ± 0.19
FRAP	1.51 ± 0.09	0.03 ± 0.05	1.03 ± 0.12	2.16 ± 0.14	0.56 ± 0.07	0.41 ± 0.03	0.10 ± 0.02	0.0002 ± 0.0003
ABTS^•^ decolorization	0.77 ± 0.05	1.30 ± 0.19	1.08 ± 0.09	4.07 ± 0.23	0.05 ± 0.04	0.12 ± 0.02	0.03 ± 0.02	0
CUPRAC	0.02 ± 0.03	0.31 ± 0.04	0.87 ± 0.11	2.07 ± 0.20	−0.01 ± 0.04	−0.02 ± 0.03	0.09 ± 0.05	0
DPPH^•^ decolorization	1.07 ± 0.13	0.35 ± 0.05	0.94 ± 0.08	0.97 ± 0.11	0.02 ± 0.02	0.04 ± 0.01	0	0
Ferricyanide reduction	0.97 ± 0.12	0.006 ± 0.010	1.25 ± 0.19	2.31 ± 0.13	0.53 ± 0.03	0.44 ± 0.08	0.59 ± 0.06	0
DCIP reduction	3.18 ± 0.21	5.35 ± 0.33	4.81 ± 0.29	4.36 ± 0.19	0	0	0	0
Methylene Blue reduction	0	See text	0	0	0	0	0	0

## Data Availability

Dataset available on request from the authors.

## Abbreviations

The following abbreviations are used in this manuscript:

ABTS	2,2′-Azinobis-(3-ethylbenzothiazoline-6-sulfonate)
CUPRAC	Cupric Ion Reducing Antioxidant Capacity
DCIP	2,6-Dichlorophenolindophenol
DPPH	2,2-Diphenyl-1-picrylhydrazyl
FRAP	Ferric Reducing Antioxidant Power
ORAC	Oxygen Radical Absorbance Capacity
TAC	Total Antioxidant Capacity
TE	Trolox Equivalent(s)
TPTZ	2,4,6-Tri(2-pyridyl)-s-triazine

## References

[1] Abeyrathne E.D.N.S., Nam K., Ahn D.U. (2021). Analytical methods for lipid oxidation and antioxidant capacity in food systems. Antioxidants.

[2] Sadowska-Bartosz I., Bartosz G. (2022). Evaluation of the antioxidant capacity of food products: Methods, applications and limitations. Processes.

[3] Silvestrini A., Meucci E., Ricerca B.M., Mancini A. (2023). Total antioxidant capacity: Biochemical aspects and clinical significance. Int. J. Mol. Sci..

[4] Özyürek M., Güçlü K., Tütem E., Başkan K.S., Erçağ E., Çelik S.E., Baki S., Leyla Y., Karaman S., Apak R. (2011). A comprehensive review of CUPRAC methodology. Anal. Meth..

[5] Işıl Berker K., Güçlü K., Tor İ., Demirata B., Apak R. (2010). Total antioxidant capacity assay using optimized ferricyanide/Prussian Blue method. Food Anal. Meth..

[6] Apak R., Güçlü K., Demirata B., Ozyürek M., Celik S.E., Bektaşoğlu B., Berker K.I., Ozyurt D. (2007). Comparative evaluation of various total antioxidant capacity assays applied to phenolic compounds with the CUPRAC assay. Molecules.

[7] Nenadis N., Lazaridou O., Tsimidou M.Z. (2007). Use of reference compounds in antioxidant activity assessment. J. Agric. Food Chem..

[8] Biskup I., Golonka I., Gamian A., Sroka Z. (2013). Antioxidant activity of selected phenols estimated by ABTS and FRAP methods. Adv. Hyg. Exp. Med..

[9] Tian X., Schaich K.M. (2013). Effects of molecular structure on kinetics and dynamics of the Trolox equivalent antioxidant capacity assay with ABTS^+•^. J. Agric. Food Chem..

[10] Skroza D., Mekinić I.G., Svilović S., Šimat V., Katalinić V. (2015). Investigation of the potential synergistic effect of resveratrol with other phenolic compounds: A case of binary phenolic mixtures. J. Food Compos. Anal..

[11] Karadag A., Ozcelik B., Saner S. (2009). Review of methods to determine antioxidant capacities. Food Anal. Meth..

[12] Pellegrini N., Vitaglione P., Granato D., Fogliano V. (2020). Twenty-five years of total antioxidant capacity measurement of foods and biological fluids: Merits and limitations. J. Sci. Food Agric..

[13] Chen Y.W.D., Santhanam K.S.V., Bard A.J. (1981). Solution redox couples for electrochemical energy storage: I. Iron (III)-iron (II) complexes with *o*-phenanthroline and related ligands. J. Electrochem. Soc..

[14] Buettner G.R. (1993). The pecking order of free radicals and antioxidants: Lipid peroxidation, α-tocopherol, and ascorbate. Arch. Biochem. Biophys..

[15] Ou B., Huang D., Hampsch-Woodill M., Flanagan J.A., Deemer E.K. (2002). Analysis of antioxidant activities of common vegetables employing oxygen radical absorbance capacity (ORAC) and ferric reducing antioxidant power (FRAP) assays: A comparative study. J. Agric. Food Chem..

[16] Prior R.L. (2015). Oxygen radical absorbance capacity (ORAC): New horizons in relating dietary antioxidants/bioactives and health benefits. J. Funct. Foods.

[17] Huang D., Ou B., Prior R.L. (2005). The chemistry behind antioxidant capacity assays. J. Agric. Food Chem..

[18] Zhuang Q.K., Scholz F., Pragst F. (1999). The voltammetric behaviour of solid 2, 2-diphenyl-1-picrylhydrazyl (DPPH) microparticles. Electrochem. Commun..

[19] Tratnyek P.G., Reilkoff T.E., Lemon A.W., Scherer M.M., Balko B.A., Feik L.M., Henegar B.D. (2001). Visualizing redox chemistry: Probing environmental oxidation–reduction reactions with indicator dyes. Chem. Educ..

[20] Vasdev S., Gill V.D., Singal P.K. (2006). Modulation of oxidative stress-induced changes in hypertension and atherosclerosis by antioxidants. Exp. Clin. Cardiol..

[21] Baranowska M., Suliborska K., Chrzanowski W., Kusznierewicz B., Namieśnik J., Bartoszek A. (2018). The relationship between standard reduction potentials of catechins and biological activities involved in redox control. Redox Biol..

[22] Farrington J.A., Land E.J., Swallow A.J. (1980). The one-electron reduction potentials of NAD. Biochim. Biophys. Acta.

[23] Han F., Song Z., Xu J., Dai M., Luo S., Han D., Niu L., Wang Z. (2021). Oxidized titanium carbide MXene-enabled photoelectrochemical sensor for quantifying synergistic interaction of ascorbic acid based antioxidants system. Biosens. Bioelectron..

[24] Genovese D., Baschieri A., Vona D., Baboi R.E., Mollica F., Prodi L., Amorati R., Zaccheroni N. (2021). Nitroxides as building blocks for nanoantioxidants. ACS Appl. Mater. Interfaces.

[25] Abramovič H., Grobin B., Poklar Ulrih N., Cigić B. (2018). Relevance and standardization of in vitro antioxidant assays: ABTS, DPPH, and Folin–Ciocalteu. J. Chem..

[26] Xie J., Schaich K.M. (2014). Re-evaluation of the 2,2-diphenyl-1-picrylhydrazyl free radical (DPPH) assay for antioxidant activity. J. Agric. Food Chem..

[27] Romanet R., Coelho C., Liu Y., Bahut F., Ballester J., Nikolantonaki M., Gougeon R.D. (2019). The antioxidant potential of white wines relies on the chemistry of sulfur-containing compounds: An optimized DPPH assay. Molecules.

[28] Goldstein S., Samuni A. (2007). Kinetics and mechanism of peroxyl radical reactions with nitroxides. J. Phys. Chem. A.

[29] Prescott C., Bottle S.E. (2017). Biological relevance of free radicals and nitroxides. Cell Biochem. Biophys..

[30] Griesser M., Shah R., Van Kessel A.T., Zilka O., Haidasz E.A., Pratt D.A. (2018). The catalytic reaction of nitroxides with peroxyl radicals and its relevance to their cytoprotective properties. J. Am. Chem. Soc..

[31] Okada Y., Tanaka K., Sato E., Okajima H. (2006). Kinetic and mechanistic studies of allicin as an antioxidant. Org. Biomol. Chem..

[32] Maldonado P.D., Alvarez-Idaboy J.R., Aguilar-González A., Lira-Rocha A., Jung-Cook H., Medina-Campos O.N., Pedraza-Chaverrí J., Galano A. (2011). Role of allyl group in the hydroxyl and peroxyl radical scavenging activity of *S*-allylcysteine. J. Phys. Chem. B.

[33] Özyürek M., Çelik S.E., Berker K.I., Güçlü K., Tor I., Apak R. (2007). Sensitivity enhancement of CUPRAC and iron (III)-phenanthroline antioxidant assays by preconcentration of colored reaction products on a weakly acidic cation exchanger. React. Funct. Polym..

[34] Ou B., Hampsch-Woodill M., Prior R.L. (2001). Development and validation of an improved oxygen radical absorbance capacity assay using fluorescein as the fluorescent probe. J. Agric. Food Chem..

[35] Benzie I.F., Strain J.J. (1996). The ferric reducing ability of plasma (FRAP) as a measure of “antioxidant power”: The FRAP assay. Anal. Biochem..

[36] Kut K., Cieniek B., Stefaniuk I., Bartosz G., Sadowska-Bartosz I. (2022). A modification of the ABTS^•^ decolorization method and an insight into its mechanism. Processes.

[37] Re R., Pellegrini N., Proteggente A., Pannala A., Yang M., Rice-Evans C. (1999). Antioxidant activity applying an improved ABTS radical cation decolorization assay. Free Radic. Biol. Med..

[38] Kuczera K., Naparło K., Soszyński M., Bartosz G., Sadowska-Bartosz I. (2023). Capsaicin toxicity to the yeast *Saccharomyces cerevisiae* is not due to oxidative stress but to disruption of membrane structure. Chem. Biol. Int..

[39] Vijayalakshmi M., Ruckmani K. (2016). Ferric reducing antioxidant power assay in plant extract. Bangladesh J. Pharmacol..

